# Differences in mental health problems in LGBT+ first year college students in Chile during the pandemic

**DOI:** 10.1007/s00127-024-02683-5

**Published:** 2024-05-31

**Authors:** Marcelo A. Crockett, Vania Martínez-Nahuel, Scarlett Mac-Ginty, Daniel Núñez, Álvaro I. Langer, Jorge Gaete

**Affiliations:** 1 Escuela de Salud Pública, Universidad de Chile, Santiago, Chile; 2 Millennium Nucleus to Improve the Mental Health of Adolescents and Youths (Imhay), Santiago, Chile; 3 Centro de Medicina Reproductiva y Desarrollo Integral del Adolescente (CEMERA), Facultad de Medicina, Universidad de Chile, Santiago, Chile; 4 Millennium Institute for Research in Depression and Personality (MIDAP), Santiago, Chile; 5 Department of Health Service & Population Research, Institute of Psychiatry, Psychology and Neuroscience, King’s College London, London, UK; 6 Centro de Investigación en Ciencias Cognitivas, Faculty of Psychology, Universidad de Talca, Talca, Chile; 7 Facultad de Psicología y Humanidades, Universidad San Sebastián, Valdivia, Chile; 8 Facultad de Educación, Universidad de los Andes. Centro de Investigación en Salud Mental Estudiantil (ISME), Santiago, Chile

**Keywords:** LGBT, Mental disorders, Suicide, Non-suicidal self-injuries, College students

## Abstract

To examine the differences in mental health problems by sexual orientation and gender identity in first-year university students in Chile during the pandemic. Methods: 7,213 first-year students aged 18 years and older from five universities participated as part of the World Mental Health - International College Student initiative in Chile. Students completed an online self-report survey between 2020 and 2021 that included measures of lifetime and 12-month major depressive episode, generalized anxiety disorder, panic disorder, bipolar disorder, drug abuse/dependence, alcohol dependence, non-suicidal self-injuries, and suicidal risk. Prevalence of mental health problems were estimated and the differences by sexual orientation and gender identity were examined using logistic and multinomial logistic regression models. Results: Between 84.1% and 98% of lesbian, gay, bisexual, trans, and other sexual and gender minority (LGBT+) students screened positive for at least one lifetime mental health problem and between 67.6% and 90.6% for two or more problems. For most outcomes, non-heterosexual (Odds Ratio [OR] between 1.25 and 7.00) and trans and gender nonconforming students (OR between 1.72 and 5.81) had significantly higher odds of positive screening for lifetime mental health problems than heterosexual and cisgender students, respectively. Similar results were observed for 12-month mental health problems. Conclusion: The results show differences in the prevalence of mental health problems in LGBT+ university students in Chile, which are consistent with those found in other countries. These results may be useful for planning interventions to improve the mental health of LGBT+ students.

**Supplementary Information:**

The online version contains supplementary material available at 10.1007/s00127-024-02683-5.

## Introduction

Mental health problems in university students are a worldwide concern due to their high prevalence [1, 2]. These have been associated with difficulties in academic performance and fulfillment of academic obligations [3, 4] and long-term negative consequences in the workplace [5, 6], relationships [7], physical health, and personal well-being [6]. Mental health problems often begin in youth [1, 8] and have high persistence into adulthood [9]. The college years are part of emerging adulthood, a period of transition from adolescence to adulthood that spans 18 to 29 years [10]. This period is characterized by a series of changes and challenges, such as entering university, the labor market, and changes in love life, which can have important implications for mental health [10, 11].

Multiple studies have indicated that lesbian, gay, bisexual, trans, and other sexual and gender minority (LGBT+) college students have a higher prevalence of mental health problems than their heterosexual and cisgender peers [12–16]. According to minority stress theory, LGBT+ individuals face specific stressors due to prejudice, discrimination, and violence directed toward sexual and gender minorities in society, which in turn have a negative effect on the mental health of LGBT+ people [17, 18]. During emerging adulthood, LGBT+ youth also face other challenges such as disclosure of sexual orientation and gender identity, gender transition, limited access to affirming health services or professionals trained in LGBT+ issues [19–21].

Several studies indicate that the recent pandemic had a negative effect on mental health, especially in young people [22]. Before the pandemic, it was estimated that one third of first-year university students experienced mental health problems during the last 12 months [1] and in Chile a sustained increase in symptoms of mental health problems had been reported [23]. Longitudinal studies with university populations indicate an increase in anxiety and mood disorders during the pandemic compared to the pre-pandemic period, especially in women and sexual and gender minorities [24]. Particularly for LGBT+ youth, the pandemic and confinement measures may have forced them to live with unsupportive family members, to hide their sexual or gender identity, to fewer opportunities to connect with people in the LGBT+ community, or to disruption of healthcare services [25–27], which may have especially affected their mental health.

There are differences in mental health outcomes within the LGBT+ group due to specific stressors. Trans people have worse health outcomes than lesbian, gay, and bisexual (LGB) people due to frequent discrimination, prejudice, and violence because of their gender identity, among other factors [28, 29]. Some studies have shown worse outcomes in bisexual people [30, 31] due to experiences of discrimination within and outside the LGBT+ community, less social visibility of bisexuality and affirmative support [31, 32]. Some studies have also observed gender differences in mental health outcomes in LGB populations. For example, in university students, lesbian and bisexual women have been reported to have a higher prevalence of mental health diagnoses than gay and bisexual men [15], and more problematic drinking, alcohol dependence, and panic disorder, but fewer depressive symptoms in lesbian women versus gay men compared to women versus heterosexual men [12]. This may account for the multiple forms of oppression (e.g., heterosexism, sexism) that disproportionately affect women and sexual minorities [33].

Most studies on mental health in LGBT+ university populations have been conducted in North American and European countries [14–16, 19, 30], with fewer studies in Latin American countries [12, 13] such as Chile [29, 34]. In the last 10 years Chile has had important legislative advances in LGBT+ issues (e.g., same-sex marriage and gender identity law). However, discrimination and violence against LGBT+ people continue to be highly prevalent problems. According to an exploratory study within the Chilean LGBT+ population, 64.3% and 49.3% of respondents reported having been discriminated against or suffered victimization in the last year, respectively, in multiple spaces (e.g., public, educational, home, social networks, among others) [35].

Currently, there is a worldwide concern to know in greater depth the health problems that affect the LGBT+ population [16, 36, 37], which can contribute to take actions to improve the mental health and well-being of this population. Having evidence in Chile is especially relevant due to the unknown extent of mental health issues among LGBT+ university students, given the existing research gap in the country [38]. Additionally, considering the potential impact of the pandemic and associated lockdown measures on this population, understanding their mental health status is of utmost importance. Therefore, the aim of this study is to examine the differences in the frequency of mental health problems by sexual orientation and gender identity in first-year university students in Chile during the pandemic.

To achieve this objective, differences based on sexual orientation were explored, encompassing individuals who are sexually, emotionally, and/or romantically attracted to persons of a different sex (heterosexuals), individuals attracted to persons of the same sex (homosexuals, also known as gays or lesbians), individuals attracted to more than one sex or gender (bisexuals), individuals who are exploring or questioning their own sexual orientation (questioning), and individuals with sexual orientations not explicitly mentioned (grouped under the category of other sexual orientations). Additionally, differences based on gender identity were investigated, including individuals whose gender identity aligns with the sex assigned to them at birth (cisgender men and cisgender women), as well as those whose gender identity diverges from their assigned sex at birth (transgender individuals and others whose gender identities do not conform to the traditional gender binary) [39].

## Methods

### Participants

The participants’ data are part of the baseline assessment of the “Longitudinal Study of Mental Health in University Students” in Chile, which is part of the World Health Organization - World Mental Health International College Student (WMH-ICS) initiative [2]. In Chile, five universities from the central and southern part of the country participated. Two universities are private and three are state-funded universities. All first-year undergraduate students from the participating universities who were at least 18 years old were invited to participate. A total of 7,225 students completed the survey. The overall response rate was 34.7%, which varied between 29.6% and 60.4% among the universities.

### Measures

The WMH-ICS initiative uses a self-report web-based survey that assesses different mental health aspects, which has been previously used in other studies of the initiative [1, 12, 13]. The variables used in this study were the following.

**Sexual orientation**. Self-report variable of sexual orientation, coded as 1 = *heterosexual*, 2 = *homosexual*, 3 = *bisexual*, 4 = *questioning*, and 5 = *other*. The category “other” groups sexual orientations not contained in the previous categories such as asexual, pansexual and demisexual.

**Gender identity**. Variable indicating the gender with which the participants identify themselves. The variable was created by crossing the variables sex assigned at birth (male or female) and current gender (man, woman, or other), where 1 = *cisgender man*, 2 = *cisgender woman*, 3 = *trans and gender nonconforming (TGNC)*. Due to the low frequency of trans men and trans women (*n* = 34), these were grouped into the TGNC category.

**Mental health problems**. The survey assesses different mental health problems using screening instruments. The mental health problems included in this study were: major depressive episode, generalized anxiety disorder, panic disorder, any bipolar disorder, and drug abuse/dependence, which were assessed using the Composite International Diagnostic Interview Screening Scales (CIDI-SC) [40]; possible alcohol dependence which was assessed using the Alcohol Use Disorders Identification Test (AUDIT) [41]; non-suicidal self-injury which was assessed using the self-report version of the Self-Injurious Thoughts and Behaviors Interview (SITBI) [42]; and suicidal risk from a modified version of the Columbia-Suicide Severity Rating Scale (C-SSRS) [43]. Mental health problems assessed with CIDI-SC used definitions and criteria based on the Diagnostic and Statistical Manual of Mental Disorders, 5th edition. Possible alcohol dependence was defined as either having a total score ≥ 16 or a total score of 8–15 plus ≥ 4 on the AUDIT dependence questions [44]. All the above mental health problems were assessed during lifetime and during the past 12 months, except for possible alcohol dependence, which was assessed only during the past 12 months. Each mental health problem corresponds to a dichotomous variable that accounts for whether a student meets the diagnostic criteria for that mental health problem (0 = *no* or 1 = *yes*), with the exception of the variable suicidal risk, which corresponds to a categorical variable that reflects the degree of suicidal risk (1 = *no risk*, 2 = *suicidal ideation only*, 3 = *planning without suicidal attempt*, 4 = *suicidal attempt*). In addition, the variables lifetime and 12-month number of problems were created, both composed of the sum of the aforementioned mental health problems of each participant. To reduce the administration time of the instrument, some mental health problems (panic disorder, bipolar disorder, alcohol dependence, and non-suicidal self-injury) were randomized, so that all participants answered the screening questions, but only a random group of 39.5–40.5% of the students answered the questionnaire for that mental health problem.

The self-report online version of the CIDI-SC in a study of the same initiative has shown adequate classification capacity (area under the curve [AUC] > 0.70), except for mania/hypomania and panic disorder (AUC between 0.57 and 0.63) compared to a diagnostic interview [45]. Another study also obtained adequate classification capacity for mood and anxiety disorders (AUC between 0.81 and 0.86) [46]. Similarly, the online self-report version of the C-SSRS has also shown good diagnostic capacity (AUC between 0.85 and 0.87) [45], as has the AUDIT (AUC between 0.78 and 0.96) [47]. The revised online version of the SITBI has been shown to have good psychometric properties and high alternate-forms reliability between the interview and the online self-report version (k = 0.84) [48].

**Sociodemographic information**. The variables age (categorized as 0 = *18–19 years* and 1 = *20 years or more*), type of university (0 = *private* and 1 = *public*) and parent’s highest level of education (transformed into a variable of three categories, where 1 = *basic or high school education*, 2 = *complete technical or incomplete university education*, 3 = *complete university and postgraduate education*) were included.

### Procedure

First, authorization was obtained from the authorities at the participating universities. Then, the project was evaluated and approved by each institution’s Scientific Ethics Committee (SEC) (SEC Facultad de Medicina, Universidad de Chile 168–2019, SEC Universidad de Talca 03-2021, SEC Universidad de los Andes CEC2021022, SEC Servicio de Salud Valdivia 075, and SEC Universidad de O’Higgins 019-2020). The survey was answered online and sent to the students’ personal e-mail addresses, which were provided by each institution. To encourage the students’ response, a communication campaign was carried out through social networks, with the support of institutional accounts. Each student gave informed consent before answering the survey. The survey lasted approximately 45 min. At the end of the survey, students received feedback on their general mental health status and information about mental health services. In addition, participants who scored at high suicidal risk were contacted for an assisted referral to a mental health service. The survey was conducted at different periods of the academic year as defined by each university. Data were collected at one university in 2020, and at all five universities in 2021 (one university participated the two years). This period was characterized by the implementation of national containment measures to prevent contagion, such as quarantines, university closures, and online classes.

### Analysis

Lifetime and 12-month prevalence of positive screening for each mental health problem were estimated by sexual orientation and gender identity. To compare prevalence by sexual orientation and gender identity, logistic regression models were used for each mental health problem, except for suicide risk, for which multinomial logistic regression was used. The category *heterosexual* was used as the reference category for sexual orientation comparisons. As for gender identity, *cisgender* (men and women) was used as the reference category. All models were adjusted for age and parents’ highest educational level, in addition to adjusting for the variables gender identity in the case of the models comparing by sexual orientation, and by sexual orientation in the models comparing by gender identity. Additionally, to examine possible gender differences among cisgender students, the effect modification of gender on the relationship between sexual orientation and mental health problems was examined. For this purpose, a multiplicative term between the variables sexual orientation transformed into dummy variables and a cisgender woman dummy variable (0 = man, 1 = woman) was incorporated in the models. Possible effect modification was examined using the Wald test with criterion *p* < 0.05 evaluating all multiplicative terms in conjunction. This analysis was only performed with cisgender students given the low frequency of TGNC students in the sample. All analyses included post-stratification weights [49] to correct for possible differences between those who completed the survey and those who did not, which were created from the sex and age variables of the entire university population under study based on university records. Multiple imputation by chained equations [50] was used to impute missing data for mental health problems that were randomized in the survey design. Analyses were performed using the Stata 17 software.

## Results

The characteristics of the participants are presented in Table 1. The final sample size used for the analyses was 7.213 students, who had complete information for the sexual orientation and gender identity variables. A third of the participants (33.6%) reported a non-heterosexual sexual orientation, mainly bisexual and questioning, and 3.3% corresponded to TGNC students. Most of the participants (77.7%) were 18–19 years old, 20.8% were 20–29 years old and only 1.5% had 30 years or more. Most of the students had parents with a complete technical or university education and were studying at a public university.

**Table 1:** Characteristics of the participants

	*n*	%
Age		
18–19	5720	77.7
20 or more	1493	22.3
Sexual orientation		
Heterosexual	4643	66.4
Homosexual	264	4.1
Bisexual	1186	15.2
Questioning	860	10.9
Other	260	3.4
Gender identity		
Cisgender man	2328	43.6
Cisgender woman	4648	53.2
TGNC	237	3.3
Parental education		
Highschool or under	2585	35.8
Technical	1605	22.5
University	2942	41.7
Type of university		
Private	1493	20.7
Public	5720	79.3

Notes. Percentages were adjusted with post-stratification weights. TGNC = Trans and gender nonconforming

Table 2 shows the prevalence of screening for mental health problems by sexual orientation and gender identity. In the total sample, the most frequent lifetime mental health problems were major depressive episode (42.2%), non-suicidal self-injury (38.4%) and suicidal ideation (24.3%), whereas 12-month problems were major depressive episode (38.4%), suicidal ideation (20.4%) and generalized anxiety disorder (18.8%). Overall, non-heterosexual and TGNC students had higher frequencies of lifetime and 12-month mental health problems compared to their heterosexual and cisgender peers, respectively. In only two mental health problems no higher frequency was observed in LGBT+ students: 12-month alcohol dependence was lower in questioning students (2.1%) than heterosexuals (3.0%) and 12-month any bipolar disorder was slightly lower in TGNC youth (4.6%) than cisgender women (4.8%), but higher than cisgender men (3.8%). Regarding the number of lifetime and 12-month mental health problems, a higher frequency of at least one mental health problem and higher frequency of co-occurrence (two or more) mental health problems were observed in non-heterosexual and TGNC students relative to heterosexual and cisgender students, respectively, especially for TGNC students. Between 84.1% and 98.0% of LGBT+ students met criteria for at least one lifetime mental health problem, and between 73.5% and 93.3% during the past 12 months. Between 67.6% and 90.6% of LGBT+ students met criteria for two or more lifetime mental health problems and between 49.1% and 76.8% during the past 12 months.

**Table 2:** Prevalence of positive screening for lifetime and 12-months mental health problems by sexual orientation and gender identity

			Sexual orientation						Gender identity		
	Total		Heterosexual	Homosexual	Bisexual	Questioning	Other		Cisgender man	Cisgender woman	TGNC
*Lifetime*											
Mayor depressive episode	42.2		35.4	55.8	58.8	50.0	59.6		33.7	47.6	67.5
Generalized anxiety disorder	19.8		16.1	27.3	29.6	22.3	32.5		12.5	24.4	43.4
Panic disorder	14.0		10.7	18.3	22.6	15.8	27.7		7.3	18.3	32.6
Any bipolar disorder	4.8		3.5	7.2	8.5	5.8	7.8		4.5	5.1	4.6
Drug abuse/dependence	11.0		9.0	15.0	17.2	10.7	19.3		12.9	8.3	30.0
Non-suicidal self-injury	38.4		29.1	50.5	61.2	51.6	60.6		27.7	44.6	79.6
Suicide											
Ideation only	24.3		24.2	22.5	19.4	32.2	24.5		23.5	25.1	20.9
Planning without attempt	22.6		18.1	33.1	33.0	27.0	36.8		20.9	23.2	34.6
Attempt	12.6		7.8	20.2	27.1	14.9	24.8		8.5	14.4	37.3
Number of problems											
0	25.3		32.5	15.9	9.0	13.0	6.5		32.0	21.2	2.0
1	20.9		23.8	14.0	13.0	19.4	13.3		25.0	18.4	7.3
2	20.3		19.1	17.4	22.3	24.6	23.8		19.3	21.4	15.5
3 or more	33.5		24.5	52.7	55.7	43.0	56.4		23.7	39.0	75.1
*12-month*											
Mayor depressive episode	38.4		31.4	49.9	55.9	46.1	56.9		29.6	44.0	63.8
Generalized anxiety disorder	18.8		15.1	25.5	28.8	21.1	31.9		11.5	23.4	42.8
Panic disorder	11.6		8.4	15.6	19.8	14.0	25.4		5.7	15.3	30.1
Any bipolar disorder	4.3		3.0	6.5	8.0	5.6	7.3		3.8	4.8	4.6
Drug abuse/dependence	7.3		5.7	10.0	11.3	8.4	13.1		9.1	5.0	21.3
Alcohol dependence	3.3		3.0	4.6	5.0	2.1	4.1		3.8	2.8	5.7
Non-suicidal self-injury	17.7		11.7	27.2	33.0	23.9	34.4		12.3	20.2	48.8
Suicide											
Ideation only	20.4		18.6	21.2	21.1	27.4	28.5		17.6	22.5	23.4
Planning without attempt	17.5		11.9	25.6	35.6	21.4	24.3		13.6	19.2	41.7
Attempt	2.5		1.6	3.9	4.1	3.1	9.6		1.6	2.8	9.8
Number of problems											
0	38.0		46.1	26.5	18.5	26.1	17.5		46.9	32.6	6.7
1	22.8		23.7	19.9	19.4	24.8	18.7		23.9	22.3	16.4
2	16.6		14.7	17.4	20.6	21.5	19.5		13.9	18.8	17.8
3 or more	22.6		15.5	36.2	41.5	27.6	44.3		15.4	26.3	59.0

Notes. Percentages were adjusted with post-stratification weights. TGNC = Trans and gender nonconforming

Table 3 show the adjusted Odds Ratios (OR) reflecting the comparison of screening for mental health problems by sexual orientation and gender identity (Supplementary Table 1 shows unadjusted OR). For most of the mental health problems examined, non-heterosexual and TGNC youth had significantly higher odds of lifetime and 12-month mental health problems than their heterosexual and cisgender peers, respectively, even after adjusting for confounding variables. Also, a higher frequency of at least one mental health problem was observed in non-heterosexual students than their heterosexual peers (lifetime OR between 2.51 and 4.67; 12-month OR between 2.12 and 3.19) and in TGNC students than their cisgender peers (lifetime OR = 6.55; 12-month OR = 4.43).

For lifetime mental health problems, especially high OR are observed for suicide planning in homosexual, bisexual, and other sexual orientation students compared to heterosexuals (OR between 3.59 and 5.67) and in TGNC students compared to cisgender students (OR = 3.53). There are also high OR for suicide attempts in non-heterosexual versus heterosexual students (OR between 3.22 and 7.00) and in TGNC versus cisgender students (OR = 5.81); as well as for non-suicidal self-injury in bisexual versus heterosexual students (OR = 3.18) and in TGNC versus cisgender students (OR = 3.71). For 12-month mental health problems, especially high OR were observed for suicidal planning in bisexual versus heterosexual (OR = 4.46) and TGNC versus cisgender (OR = 3.13) students; suicidal attempt in homosexual, bisexual, and other sexual orientation students versus heterosexual (OR between 3.21 and 6.64) and in TGNC versus cisgender students (OR = 4.07); and non-suicidal self-injury in bisexual versus heterosexual students (OR = 3.19).

No statistically significant differences (*p* > 0.05) were observed in lifetime and 12-month for any bipolar disorder in TGNC versus cisgender students, 12-month drug abuse/dependence in homosexuals versus heterosexuals, 12-month alcohol dependence in homosexuals, questioning and other sexual orientations compared to heterosexuals, and in TGNC students compared to cisgender students.

The possible effect modification of gender on the relationship between sexual orientation and mental health problems among cisgender students was ruled out, as Wald tests were non-significant overall for the multiplicative terms between sexual orientation and the dummy variable cisgender woman in each of the lifetime (*p* ≥ 0.081) and 12-month (*p* ≥ 0.135) mental health problems examined.

**Table 3:** Adjusted comparison of positive screening for lifetime and 12-months mental health problems by sexual orientation and gender identity

	Sexual orientation^1^					Gender identity^2^
	Homosexual	Bisexual	Questioning	Other		TGNC
	OR/RRR (IC 95%)	OR/RRR (IC 95%)	OR/RRR (IC 95%)	OR/RRR (IC 95%)		OR/RRR (IC 95%)
*Lifetime*						
Mayor depressive episode	2.40* (1.84–3.13)	2.27* (1.98–2.61)	1.64* (1.41–1.92)	2.06* (1.56–2.72)		1.72* (1.27–2.32)
Generalized anxiety disorder	2.02* (1.50–2.73)	1.79* (1.53–2.08)	1.25* (1.04–1.51)	1.70* (1.27–2.27)		2.20* (1.65–2.94)
Panic disorder	2.10* (1.51–2.92)	1.96* (1.64–2.34)	1.28* (1.03–1.59)	2.29* (1.68–3.12)		1.90* (1.40–2.58)
Any bipolar disorder	2.08* (1.22–3.53)	2.73* (2.04–3.65)	1.79* (1.25–2.55)	2.73* (1.66–4.50)		0.55 (0.29–1.04)
Drug abuse/dependence	1.48* (1.00–2.17)	2.36* (1.91–2.90)	1.48* (1.14–1.92)	2.05* (1.40–2.98)		2.53* (1.81–3.53)
Non-suicidal self-injury	2.59* (1.99–3.37)	3.18* (2.76–3.66)	2.18* (1.86–2.55)	2.44* (1.83–3.25)		3.71* (2.58–5.35)
Suicide^3^						
Ideation only	1.88* (1.29–2.74)	1.80* (1.47–2.22)	2.40* (1.96–2.94)	2.85* (1.81–4.48)		2.68* (1.45–4.96)
Planning without attempt	3.59* (2.54–5.08)	4.08* (3.38–4.94)	2.72* (2.20–3.36)	5.67* (3.70–8.69)		3.53* (2.00–6.25)
Attempt	5.24* (3.51–7.83)	7.00* (5.63–8.69)	3.22* (2.49–4.16)	6.91* (4.37–10.92)		5.81* (3.30–10.25)
One or more problems	2.51* (1.76–3.57)	4.15* (3.32–5.21)	2.89* (2.31–3.60)	4.67* (2.79–7.85)		6.55* (2.66–16.14)
*12-month*						
Mayor depressive episode	2.27* (1.73–2.96)	2.40* (2.09–2.76)	1.67* (1.42–1.95)	2.22* (1.67–2.94)		1.67* (1.24–2.26)
Generalized anxiety disorder	1.99* (1.46–2.72)	1.84* (1.57–2.14)	1.25* (1.03–1.52)	1.76* (1.31–2.36)		2.27* (1.69–3.04)
Panic disorder	2.24* (1.56–3.22)	2.15* (1.77–2.62)	1.44* (1.14–1.82)	2.61* (1.87–3.63)		1.99* (1.43–2.75)
Any bipolar disorder	2.20* (1.24–3.91)	2.95* (2.16–4.03)	2.00* (1.37–2.90)	2.93* (1.73–4.95)		0.59 (0.31–1.13)
Drug abuse/dependence	1.52 (0.95–2.43)	2.38* (1.85–3.06)	1.89* (1.40–2.55)	2.11* (1.36–3.29)		2.51* (1.72–3.68)
Alcohol dependence	1.45 (0.63–3.32)	1.80* (1.13–2.86)	0.73 (0.35–1.52)	1.31 (0.61–2.81)		1.42 (0.65–3.15)
Non-suicidal self-injury	2.81* (2.00–3.95)	3.14* (2.63–3.76)	2.02* (1.63–2.52)	2.76* (1.92–3.98)		2.65* (1.87–3.75)
Suicide^3^						
Ideation only	1.54* (1.05–2.26)	1.78* (1.45–2.18)	1.86* (1.51–2.28)	2.36* (1.63–3.41)		1.84* (1.13–2.98)
Planning without attempt	2.86* (2.05–4.00)	4.46* (3.72–5.36)	2.26* (1.81–2.81)	2.63* (1.76–3.91)		3.13* (2.08–4.70)
Attempt	3.21* (1.46–7.08)	3.52* (2.23–5.55)	2.25* (1.29–3.93)	6.64* (3.70–11.89)		4.07* (2.18–7.61)
One or more problems	2.38* (1.72–3.28)	3.19* (2.64–3.85)	2.12* (1.76–2.54)	2.74* (1.90–3.94)		4.43* (2.38–8.26)

Notes. 95% CI = 95% confidence intervals. OR = Odds Ratio. RRR = Relative Risk Ratio. TGNC = Trans and gender nonconforming

*p < 0.05

^1^Reference category = heterosexual. Models adjusted for age, maximum parental education, and gender identity

^2^Reference category = cisgender. Models adjusted for age, maximum parental education, and sexual orientation

^3^Multinomial logistic regression models; results expressed in RRR

## Discussion

The results of this study show that LGBT+ students have a higher frequency and co-occurrence of positive screening for different mental health problems compared to their heterosexual and cisgender peers, as well as differences in some outcomes within the LGBT+ group of students. These results contribute with evidence on the differences in the prevalence of mental health problems in LGBT+ university students in Chile, which are in line with the findings of different studies worldwide [12–16].

Mental health problems in the college population are highly prevalent, especially in the LGBT+ population. In this study it was observed that between 84.1% and 98.0%, and between 73.5% and 93.3% of the participants presented positive screening for at least one mental health problem during lifetime and 12-month, respectively, considering several mental disorders and problems such as non-suicidal self-injury and suicidal risk. Even after adjusting for confounding variables, it was observed that LGBT+ students presented significantly higher odds of positive screening for lifetime and 12-month mental health problems compared to their heterosexual and cisgender peers for most of the mental health problems examined. The higher prevalence of mental health problems in LGBT+ university population is a global health problem [12–16], which is also replicated in Chile [34]. One of the main causes of this higher prevalence is minority stress, which can have broad and severe repercussions on the mental and physical health of LGBT+ individuals [17, 18, 51, 52]. Despite progress in legislation and increased social acceptance of LGBT+ people in the world and in Chile [53], prejudice, discrimination and violence towards LGBT+ people are still a major problem in societies such as Chile [35].

The prevalence found in the overall sample stand out especially for being high compared to other reports of the same initiative, especially in 12-month major depressive episode (38.4% Chile vs. 18.5% baseline WMH-ICS) and substance use disorder (7.3% Chile vs. 3.0% baseline WMH-ICS [1]. There could be different reasons that could explain this. Prior to the pandemic, the Chilean university population already reported worrying levels of mental health problems, especially depression [54], and an increase in symptoms over the years [23]. In addition, the high frequency of depression, as well as other mental health problems found in this study could be a reflection of the effect of the pandemic on mental health, which had negative consequences especially in the young population [22]. First-year university students in 2020 and 2021 experienced major changes in their lives during the onset of the pandemic, such as university entrance, confinement measures, fear of virus infection in themselves and those close to them, changes in the education system, among others [55], which could imply an increase in stressors in this population. In addition, the social outbreak that occurred in Chile at the end of 2019 and the consequent interruption of classes for some students also adds other stressors prior to the onset of the pandemic. Furthermore, the pandemic could have had an especially greater effect on LGBT+ population [24] given specific stressors that could affect this population, such as living in an unsafe environment, hiding their identity, less social contact with other members of the LGBT+ community, among others [25–27].

Moreover, in this study, a high co-occurrence of mental health problems was observed in LGBT+ students, which was higher than their heterosexual and cisgender peers. Co-occurrence of mental health problems is common in college populations, especially in TGNC and sexual minority students [56]. Other studies have reported a higher co-occurrence in non-heterosexual versus heterosexual populations in the co-occurrence of substance use and psychological problems (mood disorders, anxiety, and psychological distress) [57, 58] which is explained in part by experiences of discrimination, victimization, and social isolation that disproportionately affect LGB individuals [58].

Mental health problems within the LGBT+ population are not homogeneously distributed. On the contrary, it has been reported that especially bisexual [31] and TGNC [28] youth have worse mental health outcomes due to specific stressors. In this study, particularly high adjusted OR were observed for the variables of suicide planning and attempt and having at least one lifetime mental health problem in bisexual, other sexual orientation, and TGNC youth. The LGBT+ population encompasses multiple sexual orientations and gender identities that share common aspects, but at the same time have specific challenges, stressors, and experiences that must be considered when analyzing and addressing mental health outcomes. On the other hand, while there are studies that point to gender differences within non-heterosexual cisgender students [12, 15], in this study gender was not observed to modify the effect of the relationship between sexual orientation and mental health problems examined among cisgender students, which could indicate that the gender differences that exist among heterosexual cisgender students are similar among non-heterosexual cisgender students in the outcomes examined.

There is ample international evidence that LGBT+ people face specific stressors resulting from a sociocultural context that has historically stigmatized and discriminated against this population [17, 18]. These minority stressors can have profound repercussions on the mental health of LGBT+ people and contribute to explain the higher prevalence of mental health problems [12–16] and the increased suicide risk in the young LGBT+ population [59]. Taking into consideration this body of evidence and the social origin of minority stressors, some authors have proposed that sexual orientation and gender identity be considered social determinants of health, which would contribute to understanding the differences in mental health observed in this population as health inequities (i.e., avoidable, remediable, and unfair differences) [60, 61]. Considering them as social determinants of health could contribute to the development and implementation of public policies in favor of health equity and social justice in this population.

One of the limitations of this study is the use of self-report screening instruments to measure mental health outcomes, which may lead to an overestimation of the real prevalence of these problems. On the other hand, although the response rate in this study (34.7%) was not lower than others studies using similar recruitment methods (between 7 and 17%) [1], the response rate obtained could reflect a selection bias, to the extent that those who answered the survey could be students with greater mental health needs or who have greater interest in these issues. To address these limitations, a communication campaign was carried out to increase the reach of the survey and encourage student participation and, on the other hand, post-stratification weights were included in the analyses to correct for possible differences between those who answered the survey and those who did not.

Globally, there is concern about the health of LGBT+ people, so there has been a call to collect data on the specific health needs of this population, as well as to carry out programs aimed at addressing these needs from a public health perspective [36, 37]. Using a large sample from five universities, this study contributes with recent and unpublished information in the Chilean context on differences in a wide range of mental health outcomes in LGBT+ university students compared to their heterosexual and cisgender peers. This contributes to the research gap in LGBT+ mental health issues in Chile and allows us to account for the magnitude of the differences in mental health in this context in a critical period for mental health, such as the pandemic. The findings of this study offer valuable insights for planning actions, interventions, and policies aimed at enhancing the mental well-being of LGBT+ college students. For instance, increasing the availability of both university-based and external mental health services can address the diverse health needs of all students, particularly those belonging to demographic groups with heightened mental health requirements. Such efforts should be coupled with initiatives aimed at mitigating barriers to accessing mental health support. Moreover, it is advisable to equip healthcare professionals with training in providing sensitive and inclusive care to LGBT+ youth, employing approaches such as affirmative care. Additionally, prioritizing prevention and early intervention strategies for these vulnerable groups, while crucial, necessitates careful attention to avoiding stigmatization and discrimination during their implementation.

It is recommended that future studies continue to generate evidence on differences in mental health outcomes by sexual orientation and gender identity, as well as delve into the intersection between LGBT+ status and other dimensions of inequity (such as rurality, ethnicity, or socioeconomic position), which could provide valuable findings for improving the well-being of this population. Also, future studies could explore the mechanisms that explain the excess of mental health problems in LGBT+ students, incorporating other types of stressors that could be important for this population (e.g., childhood adversities, recent stressors, stressors associated with college entrance, among others).

## Electronic supplementary material

Below is the link to the electronic supplementary material.

Supplementary Material 1

## References

[1] Auerbach RP, Mortier P, Bruffaerts R, WHO World Mental Health Surveys International College Student Project (2018) Prevalence and distribution of mental disorders. J Abnorm Psychol 127:623–638. 10.1037/abn0000362 30211576 10.1037/abn0000362 PMC6193834

[2] Cuijpers P, Auerbach RP, Benjet C et al (2019) The World Health Organization World Mental Health International College Student initiative: an overview. Int J Methods Psychiatr Res 28:e1761. 10.1002/mpr.1761. 30614123 10.1002/mpr.1761 PMC6590455

[3] Bruffaerts R, Mortier P, Kiekens G et al (2018) Mental health problems in college freshmen: prevalence and academic functioning. J Affect Disord 225:97–103. 10.1016/j.jad.2017.07.044 28802728 10.1016/j.jad.2017.07.044 PMC5846318

[4] Eisenberg D, Gollust SE, Golberstein E, Hefner JL (2007) Prevalence and correlates of depression, anxiety, and suicidality among university students. Am J Orthopsychiatry 77:534–542. 10.1037/0002-9432.77.4.534 18194033 10.1037/0002-9432.77.4.534

[5] Niederkrotenthaler T, Tinghog P, Alexanderson K et al (2014) Future risk of labour market marginalization in young suicide attempters—a population-based prospective cohort study. Int J Epidemiol 43:1520–1530. 10.1093/ije/dyu155 25102855 10.1093/ije/dyu155

[6] Goldman-Mellor SJ, Caspi A, Harrington H et al (2014) Suicide attempt in young people: a signal for long-term health care and social needs. JAMA Psychiatry 71:119. 10.1001/jamapsychiatry.2013.2803 24306041 10.1001/jamapsychiatry.2013.2803 PMC3946312

[7] Kerr DCR, Capaldi DM (2011) Young men’s intimate partner violence and relationship functioning: long-term outcomes associated with suicide attempt and aggression in adolescence. Psychol Med 41:759–769. 10.1017/S0033291710001182 20540815 10.1017/S0033291710001182 PMC2978767

[8] McGorry PD, Purcell R, Goldstone S, Amminger GP (2011) Age of onset and timing of treatment for mental and substance use disorders: implications for preventive intervention strategies and models of care. Curr Opin Psychiatry 24:301–306. 10.1097/YCO.0b013e3283477a09 21532481 10.1097/YCO.0b013e3283477a09

[9] Zivin K, Eisenberg D, Gollust SE, Golberstein E (2009) Persistence of mental health problems and needs in a college student population. J Affect Disord 117:180–185. 10.1016/j.jad.2009.01.001 19178949 10.1016/j.jad.2009.01.001

[10] Arnett JJ (2000) Emerging adulthood: a theory of development from the late teens through the twenties. Am Psychol 55:469–480. 10.1037/0003-066X.55.5.469 10842426

[11] Arnett JJ, Žukauskienė R, Sugimura K (2014) The new life stage of emerging adulthood at ages 18–29 years: implications for mental health. Lancet Psychiatry 1:569–576. 10.1016/S2215-0366(14)00080-7 26361316 10.1016/S2215-0366(14)00080-7

[12] Rentería R, Benjet C, Gutiérrez-García RA et al (2021) Prevalence of 12-month mental and substance use disorders in sexual minority college students in Mexico. Soc Psychiatry Psychiatr Epidemiol 56:247–257. 10.1007/s00127-020-01943-4 32886133 10.1007/s00127-020-01943-4

[13] Rentería R, Benjet C, Gutiérrez-García RA et al (2021) Suicide thought and behaviors, non-suicidal self-injury, and perceived life stress among sexual minority Mexican college students. J Affect Disord 281:891–898. 10.1016/j.jad.2020.11.038 33243555 10.1016/j.jad.2020.11.038 PMC7856251

[14] Liu CH, Stevens C, Wong SHM et al (2019) The prevalence and predictors of mental health diagnoses and suicide among U.S. college students: implications for addressing disparities in service use. Depress Anxiety 36:8–17. 10.1002/da.22830 30188598 10.1002/da.22830 PMC6628691

[15] Przedworski JM, VanKim NA, Eisenberg ME et al (2015) Self-reported mental disorders and distress by sexual orientation: results of the Minnesota College Student Health Survey. Am J Prev Med 49:29–40. 10.1016/j.amepre.2015.01.024 25997903 10.1016/j.amepre.2015.01.024 PMC4476922

[16] Brittain DR, Dinger MK (2015) An examination of health inequities among college students by sexual orientation identity and sex. J Public Health Res 4:414–414. 10.4081/jphr.2015.414 25918696 10.4081/jphr.2015.414 PMC4407041

[17] Meyer IH (2003) Prejudice, social stress, and mental health in lesbian, gay, and bisexual populations: conceptual issues and research evidence. Psychol Bull 129:674–697. 10.1037/0033-2909.129.5.674 12956539 10.1037/0033-2909.129.5.674 PMC2072932

[18] Hendricks ML, Testa RJ (2012) A conceptual framework for clinical work with transgender and gender nonconforming clients: an adaptation of the minority stress model. Prof Psychol Res Pr 43:460–467. 10.1037/a0029597

[19] Woodford MR, Kulick A, Atteberry B (2015) Protective factors, campus climate, and health outcomes among sexual minority college students. J Divers High Educ 8:73–87. 10.1037/a0038552

[20] Alessi EJ, Sapiro B, Kahn S, Craig SL (2017) The first-year university experience for sexual minority students: a grounded theory exploration. J LGBT Youth 14:71–92. 10.1080/19361653.2016.1256013

[21] Singh AA, Meng S, Hansen A (2013) It’s already hard enough being a student: developing affirming college environments for trans youth. J LGBT Youth 10:208–223. 10.1080/19361653.2013.800770

[22] Samji H, Wu J, Ladak A et al (2022) Mental health impacts of the COVID-19 pandemic on children and youth – a systematic review. Child Adoles Ment Health 27:173–189. 10.1111/camh.12501 10.1111/camh.12501 PMC8653204 34455683

[23] Álamo C, Antúnez Z, Baader T et al (2020) The sustained increase of mental health symptoms in Chilean university students over three years. Rev Latinoam Psicol 52:71–80. 10.14349/rlp.2020.v52.8

[24] Buizza C, Bazzoli L, Ghilardi A (2022) Changes in college students mental health and lifestyle during the COVID-19 pandemic: a systematic review of longitudinal studies. Adoles Res Rev 7:537–550. 10.1007/s40894-022-00192-7 10.1007/s40894-022-00192-7 PMC9362152 35966832

[25] Suen YT, Chan RCH, Wong EMY (2020) Effects of general and sexual minority-specific COVID-19-related stressors on the mental health of lesbian, gay, and bisexual people in Hong Kong. Psychiatry Res 292:113365. 10.1016/j.psychres.2020.113365 32862107 10.1016/j.psychres.2020.113365 PMC7397990

[26] Gonzales G, Loret De Mola E, Gavulic KA et al (2020) Mental health needs among lesbian, gay, bisexual, and transgender college students during the COVID-19 pandemic. J Adolesc Health 67:645–648. 10.1016/j.jadohealth.2020.08.006 32933837 10.1016/j.jadohealth.2020.08.006 PMC10251764

[27] Hawke LD, Hayes E, Darnay K, Henderson J (2021) Mental health among transgender and gender diverse youth: an exploration of effects during the COVID-19 pandemic. Psychol Sex Orientat Gend Divers 8:180–187. 10.1037/sgd0000467

[28] Su D, Irwin JA, Fisher C et al (2016) Mental health disparities within the LGBT population: a comparison between transgender and nontransgender individuals. Transgender Health 1:12–20. 10.1089/trgh.2015.0001 29159294 10.1089/trgh.2015.0001 PMC5685247

[29] Chambi-Martínez CAA, Moraga-Escobar EI, Peralta-Jiménez GA et al (2022) Social support, stress and emotional symptoms among LGBTQ + college students in Chile. Int J Sex Health 34:277–290. 10.1080/19317611.2021.2014014 38596522 10.1080/19317611.2021.2014014 PMC10903570

[30] Oswalt SB, Wyatt TJ (2011) Sexual orientation and differences in mental health, stress, and academic performance in a national sample of U.S. college students. J Homosex 58:1255–1280. 10.1080/00918369.2011.605738 21957858 10.1080/00918369.2011.605738

[31] Ross LE, Salway T, Tarasoff LA et al (2018) Prevalence of depression and anxiety among bisexual people compared to gay, lesbian, and heterosexual individuals: a systematic review and meta-analysis. J Sex Res 55:435–456. 10.1080/00224499.2017.1387755 29099625 10.1080/00224499.2017.1387755

[32] Roberts TS, Horne SG, Hoyt WT (2015) Between a gay and a straight place: bisexual individuals’ experiences with monosexism. J Bisexuality 15:554–569. 10.1080/15299716.2015.1111183

[33] Szymanski DM, Kashubeck-West S (2008) Mediators of the relationship between internalized oppressions and lesbian and bisexual women’s psychological distress. Couns Psychol 36:575–594. 10.1177/0011000007309490

[34] Valdés JM, Díaz FJ, Christiansen PM et al (2022) Mental health and related factors among undergraduate students during SARS-CoV-2 pandemic: a cross-sectional study. Front Psychiatry 13. 10.3389/fpsyt.2022.833263 10.3389/fpsyt.2022.833263 PMC9193581 35711588

[35] Subsecretaría de Prevención del Delito (2021) Estudio exploratorio de discriminación y violencia hacia personas LGBTIQ+. Resultados país. [Exploratory study of discrimination and violence towards LGBTIQ + people. Country results]. Santiago, Chile

[36] Bränström R, Van Der Star A (2013) All inclusive public health—what about LGBT populations? Eur J Public Health 23:353–354. 10.1093/eurpub/ckt054 23704057 10.1093/eurpub/ckt054

[37] Gil-Borrelli CC, Velasco C, Iniesta C et al (2017) Towards a public health system with pride: equity in health in the lesbian, gay, bisexual and trans community in Spain. Gac Sanit 31:175–177. 10.1016/j.gaceta.2016.09.013 28038788 10.1016/j.gaceta.2016.09.013

[38] Martínez V, Crockett MA, Chandra A et al (2022) State of mental health research of adolescents and youth in Chile: an ontological analysis. Int J Environ Res Public Health 19:9889. 10.3390/ijerph19169889 36011531 10.3390/ijerph19169889 PMC9408014

[39] American Psychological Association (2019) A guide for supporting trans and gender diverse students. APA Association of Graduate Students (APAGS), Washington, DC

[40] Kessler RC, Üstün TB (2004) The World Mental Health (WMH) Survey Initiative version of the World Health Organization (WHO) Composite International Diagnostic interview (CIDI). Int J Method Psychiat Res 13:93–121. 10.1002/mpr.168 10.1002/mpr.168 PMC6878592 15297906

[41] Saunders JB, Aasland OG, Babor TF et al (1993) Development of the Alcohol Use disorders Identification Test (AUDIT): WHO collaborative project on early detection of persons with harmful alcohol consumption-II. Addiction 88:791–804. 10.1111/j.1360-0443.1993.tb02093.x 8329970 10.1111/j.1360-0443.1993.tb02093.x

[42] Nock MK, Holmberg EB, Photos VI, Michel BD (2007) Self-injurious thoughts and behaviors interview: development, reliability, and validity in an adolescent sample. Psychol Assess 19:309–317. 10.1037/1040-3590.19.3.309 17845122 10.1037/1040-3590.19.3.309

[43] Posner K, Brown GK, Stanley B et al (2011) The Columbia–suicide severity rating scale: initial validity and internal consistency findings from three multisite studies with adolescents and adults. Am J Psychiatry 168:1266–1277. 10.1176/appi.ajp.2011.10111704 22193671 10.1176/appi.ajp.2011.10111704 PMC3893686

[44] Babor TF, Higgins-Biddle JC, Saunders JB, Monteiro MG (2001) The Alcohol Use disorders Identification Test: guidelines for use in primary care. World Health Organization

[45] Ballester L, Alayo I, Vilagut G et al (2019) Accuracy of online survey assessment of mental disorders and suicidal thoughts and behaviors in Spanish university students. Results of the WHO World Mental Health- International College Student initiative. PLoS ONE 14:e0221529. 10.1371/journal.pone.0221529 31487306 10.1371/journal.pone.0221529 PMC6728025

[46] Kessler RC, Calabrese JR, Farley PA et al (2013) Composite International Diagnostic interview screening scales for DSM-IV anxiety and mood disorders. Psychol Med 43:1625–1637. 10.1017/S0033291712002334 23075829 10.1017/S0033291712002334

[47] Ballester L, Alayo I, Vilagut G et al (2021) Validation of an online version of the Alcohol Use disorders Identification Test (AUDIT) for alcohol screening in Spanish university students. Int J Environ Res Public Health 18:5213. 10.3390/ijerph18105213 34068945 10.3390/ijerph18105213 PMC8156263

[48] Fox KR, Harris JA, Wang SB et al (2020) Self-injurious thoughts and behaviors Interview—Revised: development, reliability, and validity. Psychol Assess 32:677–689. 10.1037/pas0000819 32324021 10.1037/pas0000819

[49] Royal K (2019) Survey research methods: a guide for creating post-stratification weights to correct for sample bias. Educ Health Prof 2:48. 10.4103/EHP.EHP_8_19

[50] White IR, Royston P, Wood AM (2011) Multiple imputation using chained equations: issues and guidance for practice. Statist Med 30:377–399. 10.1002/sim.4067 10.1002/sim.4067 21225900

[51] Dürrbaum T, Sattler FA (2020) Minority stress and mental health in lesbian, gay male, and bisexual youths: a meta-analysis. J LGBT Youth 17:298–314. 10.1080/19361653.2019.1586615

[52] Lick DJ, Durso LE, Johnson KL (2013) Minority stress and physical health among sexual minorities. Perspect Psychol Sci 8:521–548. 10.1177/1745691613497965 26173210 10.1177/1745691613497965

[53] Ipsos (2021) LGBT + pride 2021 global survey: A 27-country Ipsos survey. New York

[54] Martínez P, Jiménez-Molina Á, Mac-Ginty S et al (2021) Mental health of higher education students in Chile: scoping review and meta-analysis. Ter Psicol 39:405–426. 10.4067/S0718-48082021000300405

[55] Tasso AF, Hisli Sahin N, San Roman GJ (2021) COVID-19 disruption on college students: academic and socioemotional implications. Psychol Trauma 13:9–15. 10.1037/tra0000996 33382329 10.1037/tra0000996

[56] Auerbach RP, Mortier P, Bruffaerts R et al (2019) Mental Disorder comorbidity and suicidal thoughts and behaviors in the. Int J Methods Psychiatr Res 28:e1752. 10.1002/mpr.1752. World Health Organization World Mental Health Surveys International College Student initiative 30450753 10.1002/mpr.1752 PMC6877246

[57] Pakula B, Shoveller J, Ratner PA, Carpiano R (2016) Prevalence and co-occurrence of heavy drinking and anxiety and mood disorders among gay, lesbian, bisexual, and heterosexual canadians. Am J Public Health 106:1042–1048. 10.2105/AJPH.2016.303083 26985615 10.2105/AJPH.2016.303083 PMC4880251

[58] Bränström R, Pachankis JE (2018) Sexual orientation disparities in the co-occurrence of substance use and psychological distress: a national population-based study (2008–2015). Soc Psychiatry Psychiatr Epidemiol 53:403–412. 10.1007/s00127-018-1491-4 29450600 10.1007/s00127-018-1491-4 PMC5862943

[59] De Lange J, Baams L, Van Bergen DD et al (2022) Minority stress and suicidal ideation and suicide attempts among LGBT adolescents and young adults: a meta-analysis. LGBT Health 9:222–237. 10.1089/lgbt.2021.0106 35319281 10.1089/lgbt.2021.0106

[60] Logie C (2012) The case for the World Health Organization’s commission on the social determinants of health to address sexual orientation. Am J Public Health 102:1243–1246. 10.2105/AJPH.2011.300599 22594723 10.2105/AJPH.2011.300599 PMC3478032

[61] Pega F, Veale JF (2015) The case for the World Health Organization’s commission on social determinants of health to address gender identity. Am J Public Health 105:e58–e62. 10.2105/AJPH.2014.302373 25602894 10.2105/AJPH.2014.302373 PMC4330845

