# Supplementary material for: Differences in mental health problems in LGBT+ first year college students in Chile during the pandemic
Source: Soc Psychiatry Psychiatr Epidemiol. 2024 May 31;59(12):2339–49. doi: 10.1007/s00127-024-02683-5 (PMC11522124; doi:10.1007/s00127-024-02683-5)
Supplement: Supplementary file 1 — Supplementary Material 1 [file 127_2024_2683_MOESM1_ESM.docx]

**Differences in mental health problems in LGBT+ first year college students in Chile during the pandemic**

Supplementary table 1. Unadjusted comparison of positive screening for lifetime and 12-months mental health problems by sexual orientation and gender identity.

|  | Sexual orientation^1^ | | | |  | Gender identity^2^ |
| --- | --- | --- | --- | --- | --- | --- |
|  | Homosexual | Bisexual | Questioning | Other |  | TGNC |
|  | OR/RRR (IC 95%) | OR/RRR (IC 95%) | OR/RRR (IC 95%) | OR/RRR (IC 95%) |  | OR/RRR (IC 95%) |
| *Lifetime* |  |  |  |  |  |  |
| Mayor depressive episode | 2.31* (1.79, 2.99) | 2.61* (2.28, 2.99) | 1.83* (1.57, 2.13) | 2.70* (2.07, 3.51) |  | 2.95* (2.21, 3.93) |
| Generalized anxiety disorder | 1.96* (1.47, 2.61) | 2.19* (1.89, 2.55) | 1.50* (1.25, 1.80) | 2.52* (1.91, 3.32) |  | 3.27* (2.49, 4.29) |
| Panic disorder | 1.87* (1.36, 2.57) | 2.43* (2.06, 2.88) | 1.57* (1.27, 1.94) | 3.20* (2.39, 4.30) |  | 3.15* (2.36, 4.20) |
| Any bipolar disorder | 2.12* (1.26, 3.55) | 2.54* (1.92, 3.35) | 1.68* (1.19, 2.37) | 2.30* (1.40, 3.78) |  | 0.95 (0.51, 1.79) |
| Drug abuse/dependence | 1.80* (1.24, 2.59) | 2.11* (1.74, 2.55) | 1.22 (0.94, 1.57) | 2.42* (1.71, 3.44) |  | 3.69* (2.72, 5.01) |
| Non-suicidal self-injury | 2.49* (1.93, 3.22) | 3.84* (3.35, 4.41) | 2.60* (2.23, 3.03) | 3.75* (2.87, 4.90) |  | 6.64* (4.72, 9.35) |
| Suicide^3^ |  |  |  |  |  |  |
| Ideation only | 1.92* (1.32, 2.79) | 1.96* (1.60, 2.40) | 2.58* (2.12, 3.14) | 3.65* (2.35, 5.68) |  | 4.89* (2.67, 8.93) |
| Planning without attempt | 3.77* (2.67, 5.32) | 4.46* (3.70, 5.37) | 2.88* (2.34, 3.54) | 7.32* (4.83, 11.09) |  | 8.89* (5.05, 15.64) |
| Attempt | 5.35* (3.62, 7.90) | 8.49* (6.90, 10.45) | 3.69* (2.87, 4.75) | 11.44* (7.33, 17.84) |  | 18.07* (10.26, 31.80) |
| One or more problems | 2.55* (1.80, 3.62) | 4.87* (3.90, 6.08) | 3.23* (2.60, 4.02) | 6.89* (4.14, 11.45) |  | 17.19* (6.96, 42.42) |
|  |  |  |  |  |  |  |
| *12-months* |  |  |  |  |  |  |
| Mayor depressive episode | 2.17* (1.68, 2.82) | 2.77* (2.42, 3.17) | 1.87* (1.60, 2.18) | 2.88* (2.21, 3.76) |  | 2.94* (2.21, 3.90) |
| Generalized anxiety disorder | 1.93* (1.43, 2.59) | 2.27* (1.95, 2.64) | 1.51* (1.25, 1.82) | 2.64* (1.99, 3.49) |  | 3.40* (2.58, 4.48) |
| Panic disorder | 2.03* (1.44, 2.87) | 2.71* (2.25, 3.27) | 1.79* (1.42, 2.25) | 3.73* (2.73, 5.09) |  | 3.48* (2.57, 4.73) |
| Any bipolar disorder | 2.24* (1.29, 3.89) | 2.79* (2.07, 3.75) | 1.90* (1.33, 2.73) | 2.53* (1.50, 4.28) |  | 1.07 (0.57, 2.01) |
| Drug abuse/dependence | 1.82* (1.16, 2.86) | 2.08* (1.65, 2.63) | 1.51* (1.13, 2.02) | 2.47* (1.62, 3.75) |  | 3.70* (2.61, 5.25) |
| Alcohol dependence | 1.53 (0.67, 3.48) | 1.68* (1.08, 2.62) | 0.66 (0.32, 1.37) | 1.38 (0.68, 2.79) |  | 1.78 (0.87, 3.68) |
| Non-suicidal self-injury | 2.82* (2.02, 3.95) | 3.73* (3.13, 4.45) | 2.37* (1.92, 2.92) | 3.97* (2.83, 5.55) |  | 4.79* (3.45, 6.65) |
| Suicide^3^ |  |  |  |  |  |  |
| Ideation only | 1.57* (1.08, 2.27) | 1.96* (1.61, 2.40) | 2.07* (1.69, 2.53) | 2.76* (1.95, 3.90) |  | 2.80* (1.78, 4.40) |
| Planning without attempt | 2.97* (2.13, 4.13) | 5.20* (4.36, 6.22) | 2.54* (2.05, 3.15) | 3.70* (2.55, 5.36) |  | 6.08* (4.14, 8.91) |
| Attempt | 3.31* (1.54, 7.12) | 4.43* (2.88, 6.82) | 2.70* (1.58, 4.59) | 10.86* (6.19, 19.07) |  | 10.60* (5.87, 19.12) |
| One or more problems | 2.38* (1.73, 3.27) | 3.77* (3.14, 4.54) | 2.42* (2.03, 2.89) | 4.04* (2.85, 5.73) |  | 8.98* (4.86, 16.59) |

Notes. 95% CI = 95% confidence intervals. OR = Odds Ratio. RRR = Relative Risk Ratio. TGNC = Trans and gender nonconforming.

**p* < 0.05.

^1^Reference category = heterosexual.

^2^Reference category = cisgender.

^3^Multinomial regression models; results expressed in RRR.
